# Combating the conservative practices: block chain technology for securing electronic health records in remote medical systems

**DOI:** 10.1097/MS9.0000000000004866

**Published:** 2026-03-16

**Authors:** Waseem Sajjad, Sarmad Javed, Uzma Khan, Shamaim Naeem, Ihsan Qamar

**Affiliations:** a Department of Medicine, King Edward Medical University, Mayo Hospital, Lahore, Pakistan; b Department of Medicine, Peshawar Medical College, Peshawar, Pakistan; c Faculty of Medicine, Spinghar University, Kabul, Afghanistan

**Keywords:** blockchain technology, confidentiality of patients’ data, electronic health care records (EHRs), patients’ privacy, public health, remote health care systems

## Abstract

The need for protecting electronic health records (EHRs) in distant medical systems is discussed in the article, which also suggests using blockchain technology as a viable countermeasure to more conventional, less secure practices. The current approaches to managing EHRs in remote health care are frequently vulnerable to hacks and data theft, which impedes the advancement of telemedicine. Contrarily, due to its decentralized and immutable nature, blockchain technology offers greater security, guaranteeing patient data are kept private and unaltered. Additionally, it makes it easier for health care systems to communicate data seamlessly, improving patient care. For widespread adoption, however, issues like scalability and infrastructure needs must be resolved. An ecosystem for remote health care that is more secure and patient-focused can result from embracing blockchain-based EHRs.

## Manuscript

Electronic health records (EHRs) security in remote medical systems is an important concern, and it could potentially be possible for blockchain technology to replace these established practices. Traditional EHR administration strategies typically fall short of providing the necessary degree of confidentiality and availability for remote health care services, leading to compromising patient data and obstructing the advancement of telemedicine. The perks and limitations of blockchain technology for safeguarding EHRs are some inevitable considerations in this era to be well understood.

When it comes to maintaining data security and privacy, traditional ways of administering EHRs in remote medical systems have faced significant difficulties. The susceptibility of centralized systems to cyberattacks as well as data theft puts sensitive patient data at risk^[^1^]^. Additionally, the fragmentation of medical information due to a lack of seamless integration between different health care systems makes it challenging to effectively coordinate care and leads to subpar treatment outcomes^[^2^]^. These issues make it less likely for patients to fully embrace options for remote health care, and medical professionals often hesitate to use these kinds of technology out of worries about data integrity.

On the contrary, the security of EHRs has benefited from various breakthroughs in blockchain technology. Blockchain eliminates the risk of unwanted access or tampering by using a decentralized and distributed ledger to ensure that patient data are not kept in a single, susceptible location^[^3^]^. Each transaction that is added to the blockchain is encrypted and connected to earlier transactions to establish an unchangeable chain of data. Patients are more confident as a result of the openness and immutability of the system, which gives them more control over who can access their medical records and guarantees that their privacy is preserved^[^4^]^.

Blockchain technology also makes it easier for different health care systems and providers to communicate with one another. Even from bigger health care networks, this smooth data exchange enables remote medical facilities to access vital patient information in real-time, which may be essential in emergencies or when treating patients with complicated medical histories^[^5^]^. Blockchain-based EHRs’ improved interoperability and accessibility open the door for a more effective and patient-focused distant health care ecosystem.

However, blockchain technology is not without its drawbacks. Especially in remote areas with limited resources, deployment of this technology necessitates major infrastructure construction and expenditure. Additionally, scalability is still an issue since as transaction volumes rise, blockchain networks may become slower and more resource intensive^[^6^]^. For blockchain-based EHRs to be widely used in distant medical systems, these difficulties must be overcome.

In conclusion, it should be noted that the security of EHRs in distant medical systems is a serious issue that necessitates creative solutions. A more secure and patient-focused remote health care ecosystem is possible thanks to the development of blockchain technology. Blockchain-based EHRs have the potential to transform remote health care services and improve patient outcomes by providing improved security, transparency, and interoperability^[^7^]^.

This work has been reported in accordance with TITAN Guidelines^[^8^]^.

## Data Availability

Already provided. (Any queries regarding it will be entertained with pleasure.)
